# Evaluation of *Streptococcus* species isolated from subclinical sheep mastitis by molecular methods and determination of virulence factors and antimicrobial resistance genes

**DOI:** 10.17221/42/2023-VETMED

**Published:** 2023-09-25

**Authors:** Volkan Ozavci, Hafize Tugba Yuksel Dolgun, Sukru Kirkan, Yigit Seferoglu, Zeynep Semen, Ugur Parin

**Affiliations:** ^1^Department of Microbiology, Faculty of Veterinary Medicine, Dokuz Eylul University, Izmir, Turkiye; ^2^Department of Microbiology, Faculty of Veterinary Medicine, Aydin Adnan Menderes University, Aydin, Turkiye; ^3^Department of Biochemistry, Faculty of Veterinary Medicine, Dokuz Eylul University, Izmir, Turkiye

**Keywords:** dairy sheep, mastitis, PCR, Streptococcus strains

## Abstract

*Streptococcus* (*S.*) species are important pathogens that cause mastitis in sheep. The study aimed to examine *Streptococcus* species in sheep milk with subclinical mastitis, assessing their prevalence, antimicrobial resistance, and virulence genes. A total of 200 milk samples were collected from sheep farms in İzmir’s five districts. Out of 32 (28.6%) *Streptococcus* isolates identified by phenotypic methods, 25 were genotypically identified as *S. uberis*, 5 as *S. agalactiae*, and 2 as *S. dysgalactiae*. Disk diffusion was used to determine the antimicrobial resistance of the isolates. PCR was employed to identify antimicrobial resistance and virulence genes in the isolates. The highest resistance was found for cloxacillin (100%), and the highest sensitivity was found for florfenicol (84%). The most common resistance gene combination was *tetM*+*tetS* (3/32) for *S. uberis* in 9.4%. A total of five virulence genes were detected. *GapC*+*sua* (56.2%) constituted the most common gene pattern. The highest virulence gene *gapC* was detected in 78.1% (25/32) of the isolates. The *cylE* gene was not detected (0%) in the isolates. *Streptococcus* species may play a role in mastitis in sheep, emphasising the need for meticulous hygienic milking practices.

Mastitis is an inflammation of the mammary tissue due to factors such as care, milking, breed, age, feeding, changes in environmental conditions, and microbiological factors (Zigo et al. 2021). Staphylococci, streptococci, mycoplasmas, and coliforms are species that etiologically contribute to widespread udder diseases. Streptococci are Gram-positive spherical bacteria (0.5–2 μm) that typically form pairs or chains. They are classified based on the Lancefield group taxonomic system, which considers colony morphology, haemolysis, and serological specificity (Ruegg 2017; Kabelitz et al. 2021). *Streptococcus* (*S.*) *uberis* (Vezina et al. 2022), *Streptococcus dysgalactiae* (Guerreiro et al. 2013), and *Streptococcus agalactiae* are the most common species causing mammary gland infections. Among microorganisms responsible for sheep mastitis, streptococci rank second in importance after staphylococci (Kumar et al. 2013). Both *S. uberis* and *S. agalactiae* can induce chronic mastitis in cows (An et al. 2021). Although, *S. uberis* is primarily an environmental pathogen, cases of transmission have been observed. It typically exhibits gamma haemolysis (i.e., no haemolysis) on blood agar, though it can also display alpha-haemolytic behaviour in some instances. Its identification can be confirmed through a variable CAMP phenotype test and degradation of esculin, sodium hippurate, and inulin (Kabelitz et al. 2021). *S. dysgalactiae* is a pathogen that can survive in both the host and the environment. While some strains are alpha-haemolytic, most are non-haemolytic. It is phenotypically CAMP-negative and does not degrade esculin (Wente and Kromker 2020; Kabelitz et al. 2021). Several potential virulence factors have been identified for *S. uberis*, including sua, which aids in mammary epithelial cell adhesion and invasion, and gapC, a surface dehydrogenase protein (Kerro Dego et al. 2021). As a major mastitis-causing pathogen, *S. agalactiae* possesses virulence factors like the CAMP factor *cfb* and the toxin *cylE* (Zastempowska et al. 2022). Furthermore, *S. agalactiae* is capable of transferring genetic material to other mastitis pathogens, such as *S. uberis* and *S. dysgalactiae*, through genes like *napr* and *eno*-binding host plasminogen protein, thereby contributing to host infection and colonization. The adherence of plasminogen on the bacterial surface plays a pivotal role in the pathogenic mechanism of bacterial adhesion to host cells (Shen et al. 2021). However, antibiotic resistance is also a contributing factor to the failure of mastitis treatment. *S. dysgalactiae* isolates were found to carry *blaZ*, *ermA*, *ermB*, *ermC*, and *lnuA* genes, while *S. agalactiae* isolates were found to harbour *blaZ*, *ermB*, *ermC*, *lnuA*, *tetK*, *tetL*, and *tetM* genes (Ahmed et al. 2020). Streptococcal isolates from mastitis sheep milk confirmed via PCR were found to contain genes such as *ermB*, *ermC*, *linB*, and antibiotic resistance genes like *tetM* and *tetO* (Saed and Ibrahim 2020). Aminoglycoside resistance in streptococci is also mediated by genes like *aad-6* and *aphA-3*, which lead to enzymatic inactivation of antibiotics.

The countries with the world’s largest sheep populations include China, India, Australia, Nigeria, Iran, Ethiopia, and Türkiye (Sevinc et al. 2022). In Türkiye, the sheep population averages 46 million, and the country produced approximately 1 100 000 tons of sheep’s milk in 2022. According to the Governor’s office (MAF 2023), Izmir province and its districts have an average of 672 000 sheep, with around 320 000 animals being milked. Raw sheep milk production decreased by 6.7% in 2022 compared to the previous year (TUIK 2022). Although sheep milk production seems to be declining according to TUIK data, our region plays an important role in the country’s sheep milk production. Therefore, investing in academic research on animal husbandry with the aim of enhancing and contributing to the nation’s sheep milk production could yield positive outcomes for effective animal husbandry practices.

This study aimed to identify multiple antibiotic-resistant *Streptococcus* species in milk collected from sheep with subclinical mastitis in the Küçük Menderes Basin (including Bayındır, Beydağı, Kiraz, Ödemiş, and Tire) in Izmir province. The study aimed to assess the susceptibility of isolated *Streptococcus* species to antibiotics and identify the presence of virulence genes such as *gapC*, *sua*, *cylE*, *cfb*, *eno*, and *napr*. This would provide better insights into the potential pathogenicity of these bacteria and inform effective treatment strategies for mastitis. Additionally, the study revealed antibiotic resistance genes including *lnuD*, *ermB*, *ermC*, *tetL*, *tetS*, *aad-6*, and *blaZ*.

## MATERIAL AND METHODS

### Sample collection

A total of 200 milk samples were collected from 100 sheep of various breeds and ages in selected dairy farms in the Küçük Menderes Basin between November and February 2022. These milk samples were collected from sheep suspected of having mastitis based on the anamnesis provided by veterinarians or technicians from farms in the districts of Kiraz, Tire, Ödemiş, Bayındır and Beydağı districts. Samples of sheep’s milk were collected in equal quantities in each district. Teats were cleaned and disinfected with 75% ethanol before collecting milk samples. The first foremilk was discarded and 200 milk samples were collected aseptically into sterile tubes containing 10–15 ml of milk. California mastitis test (CMT) was applied to the collected milk samples and the results were evaluated as –, +, ++ and +++. According to the CMT results, 88 samples were considered negative, and 112 samples were considered positive. The investigation was continued on 112 CMT-positive samples. The results of the CMT applied to milk were evaluated according to the method of Schalm et al. (1971). CMT-positive samples were kept under cold chain conditions and transported to the molecular laboratories of Aydin Adnan Menderes University, Faculty of Veterinary Medicine, Department of Microbiology for laboratory analysis.

### Isolation and identification *Streptococcus* strains

To analyse the milk samples, each sample was inoculated on Columbia blood agar (Thermo Fisher Scientific, Waltham, MA, USA) medium with 5% defibrinated sheep blood. The agar plates were aerobically incubated at 37 °C for 24–48 hours. The analysis included the assessment of phenotypic and biochemical colony morphology, microscopic features, Gram stain, vancomycin susceptibility, haemolysis on blood agar, esculin hydrolysis, catalase, oxidase, PYR (pyrrolidonyl arylamidase), CAMP (Christie–Atkins–Munch-Petersen), and isolation of *Streptococcus* strains. Moreover, a selective medium, Edward’s medium supplemented with 6% defibrinated sheep blood, was used to evaluate esculin hydrolysis for *S. uberis*, *S. dysgalactiae*, and *S. agalactiae* (Kaczorek et al. 2017a).

### DNA extraction from *Streptococcus* strains

The *Streptococcus* isolates identified through biochemical methods such as Gram staining, catalase test, and haemolysis patterns were suspended in 500 μl of sterile distilled water. Subsequently, a genomic DNA extraction kit (Hibrigen^®^, Kocaeli, Türkiye) was employed as per the manufacturer’s protocol to extract DNA from the prepared *Streptococcus* suspensions. The obtained *Streptococcus* DNAs were then stored in a deep freezer at –20 °C for further molecular studies such as PCR and sequencing.

### Molecular identification of *Streptococcus* species

For PCR, the final concentration for each sample was 10X *Taq* enzyme buffer solution (1X), 50 mM MgCl_2_ (2 mM), 10 mM dNTP (0.2 mM), 40 ρmol of each primer, and 1.5 U/μl of *Taq* DNA polymerase (Thermo Fisher Scientific, Waltham, MA, USA). The reaction was performed in a volume of 25 μl with 3 μl of DNA added per sample. Primers and cycling conditions were used as defined (Kaczorek et al. 2017a). PCR products were visualised by electrophoresis of 10 μl of each sample on a 2% agarose gel at 80 V for 60 min, and the results were evaluated using a gel documentation system (Vilber Lourmat, Collégien, France).

### Sanger sequence typing of identified *Streptococcus* species

The confirmation of *Streptococcus* isolates by Sanger sequencing used Universal primers 357F (5'-CTCCTACGGGGAGGCAGCAG-3') and 1 492R (5'-GTTACCTTGTTACGACTT-3'). The previously described protocol for PCR analysis was applied to the isolates (Turner et al. 1999). PCR products were purified using ExoSAP-IT^TM^ (GML^®^) PCR Product Cleanup Reagent (Thermo Fisher Scientific, Waltham, MA, USA), followed by Sephadex (GML^®^) (Sigma-Aldrich, St. Louis, MO, USA) for sequence PCR product purification. Sequencing of purified PCR products was performed using the GenomeLab Dye Terminator Cycle Sequencing (DTCS) Quick Start Kit and the Sanger Sequence instrument (Beckman Coulter, Inc., Fullerton, CA, USA). Standard Nucleotide BLAST^®^ NCBI Genomic Reference Sequences were used to analyse the nucleotide sequences of the PCR products.

### Detection of virulence genes of *Streptococcus* species

The multiplex PCR method was used to examine the presence of virulence-related genes in all strains that were phenotypically identified as *S. uberis*, *S. dysgalactiae*, and *S. agalactiae*. The preparation of the master mix involved adding 20 ng of DNA sample to a total volume of 50 μl, with 1 μM of each primer, 0.4 mM dNTP, 1.5 mM MgCl_2_, 1 × reaction buffer, and 1.5 U/μl *Taq* DNA polymerase (Thermo Fisher Scientific, Waltham, MA, USA).

The primer sequences, product sizes, and annealing temperatures special to virulence-related target genes were used in our study to detect *S. uberis*, *S. agalactiae*, and *S. dysgalactiae* (Kaczorek et al. 2017b).

### Determination of antimicrobial susceptibility of *Streptococcus* strains

Mueller-Hinton agar supplemented with 5% sheep blood was used in the disk diffusion method, and the results were assessed following incubation of the media at 37 °C for 48 hours. The antimicrobial susceptibility results were interpreted according to the standards set by the European Committee on Antimicrobial Susceptibility Testing (EUCAST 2013) for amoxicillin (25 μg), florfenicol (30 μg), neomycin (30 μg), and oxytetracycline (30 μg) discs, and by the Clinical and Laboratory Standards Institute (CLSI 2015) for ampicillin (25 μg), amoxicillin + clavulanic acid (30 μg), cefoperazone (75 μg), ciprofloxacin (5 μg), cloxacillin (5 μg), erythromycin (15 μg), gentamicin (10 μg), and penicillin G (6 μg) discs.

### Detection of antimicrobial resistance genes from *Streptococcus* strains by using PCR

The resistance genes for lincosamide (*lnuD*) (Haenni et al. 2011), macrolide (*ermC* and *ermB*) (Bingen et al. 2000), tetracycline (*tetL*, *tetM*, and *tetS*) (Ng et al. 2001), aminoglycoside (*aad-6*) (Zhang et al. 2018), and penicillin (*blaZ*) (Kot et al. 2020) were determined by PCR using the primers as described. In addition, we utilised strain-specific primers targeting 16S rRNA and *hsp40* to validate the *S. agalactiae*, *S. dysgalactiae,* and *S. uberis* strains, respectively (Kaczorek et al. 2017a).

## RESULTS

**Figure 1:**
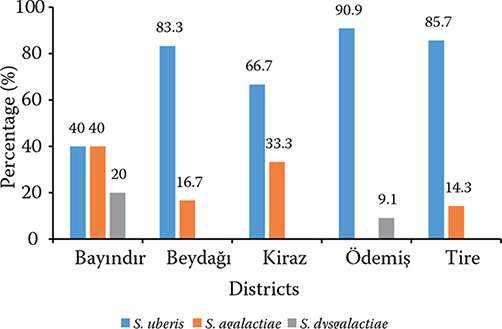
Isolation percentages in districts for *Streptococcus* isolates

As a result of microbiological phenotypic analyses of 112 (56%) CMT-positive subclinical mastitis sheep milk samples, 32 (28.6%) *Streptococcus* species were isolated. The isolates were typed through Gram staining, culture characteristics, biochemical tests, and 16S rRNA Sanger sequencing. Of the 32 isolates, 25 (78.1%) were identified as *S. uberis*, 5 (15.6%) as *S. agalactiae*, and 2 (6.3%) as *S. dysgalactiae*. The distribution of *Streptococcus* infection percentages per district is in Figure 1.

**Figure 2:**
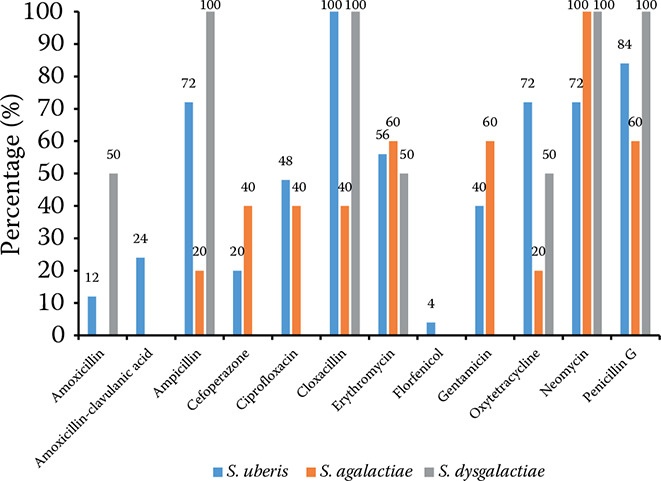
Resistance percentages for *Streptococcus uberis* (*S. uberis*)*, Streptococcus agalactiae* (*S. agalactiae*) and *Streptococcus dysgalactiae* (*S. dysgalactiae*) strains from milk samples with mastitis

Antimicrobial susceptibility testing showed that the 32 *Streptococcus* isolates from mastitis-affected milk samples demonstrated varying degrees of resistance to 12 antimicrobial agents (Figure 2). All isolates exhibited resistance to 3 or more antimicrobial agents. Among the 25 *S. uberis* isolates, 19 (76%) were multiresistant to 5–9 antimicrobial agents, while 1 (4%) was multiresistant to 11 antimicrobial agents. Cloxacillin exhibited the highest resistance rate (100%), followed by penicillin G (84%), ampicillin, oxytetracycline, and neomycin (72%). Among the 5 *S. agalactiae* isolates, 2 (40%) were multiresistant to 7–8 antimicrobial agents, and among the 2 *S. dysgalactiae* isolates, 2 (50%) were multiresistant to 4–7 antimicrobial agents. *S. uberis* exhibited resistance to cloxacillin (100%) and penicillin G (84%). *S. agalactiae* displayed the highest resistance rate to neomycin (100%), and *S. dysgalactiae* had the highest resistance rate to ampicillin, cloxacillin, neomycin, and penicillin G (100%). Florfenicol had the highest susceptibility rate (100%) among antimicrobials for *S. agalactiae*, and both florfenicol and gentamicin had the highest susceptibility rate (100%) for *S. dysgalactiae*. Florfenicol had the susceptibility rate 84% and an intermediate susceptibility of 12% for *S. uberis*.

**Figure 3:**
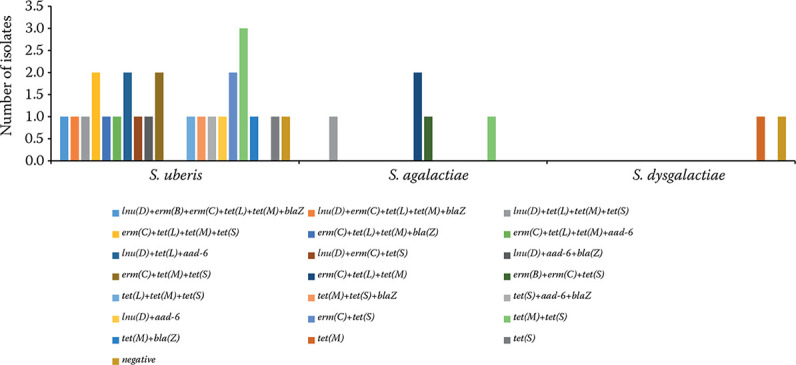
Distribution of resistance genes and number of *Streptococcus* strains isolated from mastitis milk samples

Antimicrobial resistance genes were detected in 30 (93.8%) *Streptococcus* isolates (Figure 3). Most of the strains contained at least one antibiotic resistance gene. Resistance genes for lincosamides (*lnuD*), macrolides (*ermB*, *ermC*), tetracyclines (*tetL*, *tetM*, *tetS*), aminoglycosides (*aad-6*), and penicillins (*blaZ*) were tested by PCR in all isolates. A total of nineteen resistance gene combination patterns were found, with *tetM* and *tetS* resistance genes being the most common. The *tetM*+*tetS* combination was found in 3/32 (9.4%) *S. uberis* isolates. In *S. agalactiae*, 2/32 (6.3%) of the isolates carried *ermC*+*tetL*+*tetM* gene combination. Only the tetracycline-related *tetM* gene was detected in *S. dysgalactiae* 1/32 (3.1%). The presence of different classes of antimicrobial-related genes in the isolates was also calculated. Lincosamide resistance genes (*lnuD*) were detected in nine (28.1%) isolates, macrolide resistance genes [*ermB* (2), *ermC* (10)] in 14 isolates (43.8%), tetracycline resistance genes [*tetM* (15), *tetS* (12), *tetL* (10)] in 28 isolates (87.5%), aminoglycoside resistance genes (*aad-6*) in six isolates (18.7%), and penicillin resistance genes (*blaZ*) in seven isolates (21.9%). Two samples (6.3%) did not show any antibiotic resistance genes.

In this study, upon examining the virulence genes of the identified *Streptococcus* isolates, it was found that the *gapC*+*sua* genes were positive in 18 (56.2%) *S. uberis* isolates, while only the *gapC* gene was positive in 7 (21.8%) *S. uberis* isolates. *Eno* and *napr* genes (6.2%) were positive in *S. dysgalactiae* (*n* = 2) isolates. The *cylE* gene was negative in all isolates.

**Figure 4:**
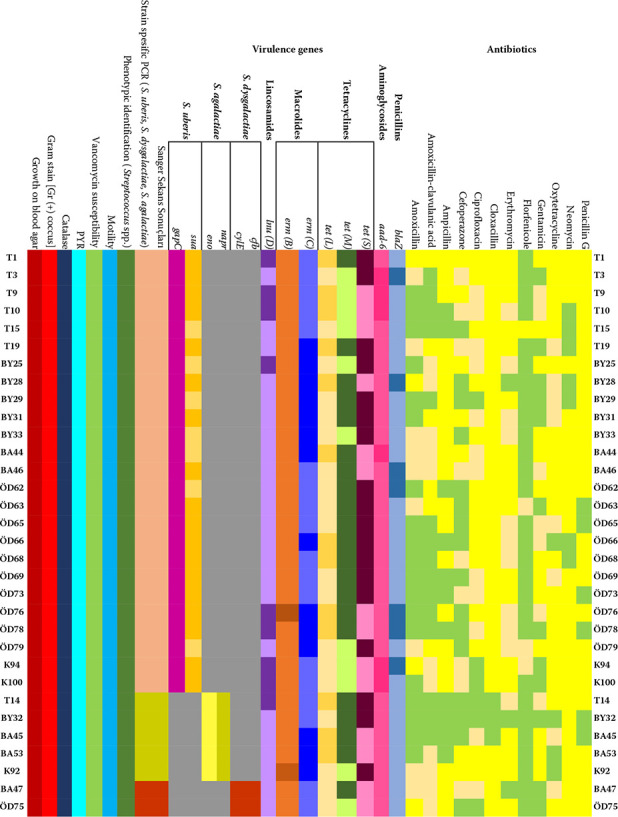
Heatmap; all results for *Streptococcus uberis* (*S. uberis*)*, Streptococcus agalactiae* (*S. agalactiae*), *Streptococcus dysgalactiae* (*S. dysgalactiae*) isolated from sample of milk mastitis (*n* = 32) In the virulence gene parameters, dark colours represent positive values, and light colours represent negative values. The yellow colour represents resistant values, the beige colour represents intermediate values and the green colour represents susceptible values for antimicrobial activity

In addition, all of the phenotypic isolation, 16S rRNA PCR, Sanger sequence, virulence genes, antimicrobial resistance genes and antibiogram results obtained in the study are given in the heatmap (Figure 4).

## DISCUSSION

The incidence of clinical mastitis in sheep flocks sampled worldwide is relatively low (1.2–3%), but significant variation can exist within flocks (0–37%) (Murphy et al. 2018). Subclinical mastitis (SCM) lacks visual symptoms, but diagnosis is possible through bacterial culture and/or quantification of somatic cell count (SCC) in milk. The morbidity rate of SCM is much higher in sheep than in clinical mastitis (CM) (12–50%) (Murphy et al. 2018). *S. uberis* and *S. dysgalactiae* are opportunistic environmental animal pathogens, while *S. agalactiae* is a primary pathogen causing contagious disease (Chen et al. 2021). The incidence of clinical mastitis in sheep has generally been reported to be less than 5%, with the incidence of subclinical cases ranging from 16% to 35% (Guerreirot al. 2013). In another study, the incidence of *Streptococcus* strains in mastitis sheep milk was found to be 8% (Ceniti et al. 2017). We analysed 200 subclinical mastitis sheep milk samples and isolated *Streptococcus* spp. in 32 (28.6%). In addition, studies have reported the following species ratios 5.1% and 2.1% for *S. uberis* and *S. dysgalactiae*, respectively (Ceniti et al. 2017), and 19% for *S. agalactiae* (Abd-Elfatah et al. 2023). Rosa et al. (2022) reported that the most common streptococcal species in sheep and goats with mastitis were *S. uberis* (89.5%) and *S. dysgalactiae* (3.5%). In our study, *S. agalactiae* (15.6%) and *S. dysgalactiae* (6.3%) were detected at low percentages, but this was consistent with these findings. In contrast, *S. uberis* was detected at a higher rate (78%). This raises the question of whether there is a similar risk in sheep, as has been suggested for goats, where mechanical milking has been associated with a higher risk of bacterial positivity and *S. uberis* infection (Novac and Andrei 2020).

There are few studies on the detection of virulence genes in *Streptococcus* infections in sheep. When considering the virulence genes for *Streptococcus* strains isolated from clinical cases of mastitis in dairy cattle, a study reported a wide frequency of the *cfb* gene (93%) and *cylE* gene (90.6%) in *S. agalactiae* (Kaczorek et al. 2017a). Shi et al. (2023) reported that the *cfb* gene encodes the haemolysis-promoting factor CAMP and that this gene is one of the causative factors of *Streptococcus* infection. However, in our study on subclinical sheep mastitis, these genes were detected only in *S. dysgalactiae* species. Kaczorek et al. (2017a) reported the presence of *sua* (96%) and *gapC* (98%) genes in *S. uberis*, and *eno* (76%) and *napr* (83%) genes in *S. dysgalactiae*. All our positive isolates showed at least one virulence gene, with the *sua* and *gapC* genes being the most frequently detected. These results align with existing research on virulence-associated genes. Furthermore, we found that the *eno* and *napr* genes were comparatively less prevalent than other genes, suggesting a potential role in *S. dysgalactiae* infection in dairy sheep. Notably, the *cylE* gene was not detected.

The characterisation of isolates is important to gather information on resistance and to optimise therapy. Pathogenic *Streptococcus* isolates causing mastitis in dairy cows can exhibit severe resistance to erythromycin and penicillin (Tian et al. 2019). El-Shafei et al. (2020) reported that *S. dysgalactiae* strains (87%) demonstrated higher resistance to tetracycline than *S. agalactiae* strains (37%) in cows and buffaloes. Additionally, both strains were susceptible to penicillin and amoxicillin. In contrast, we observed a high rate of penicillin resistance in *S. uberis* and *S. dysgalactiae*, while *S. agalactiae* displayed high sensitivity*. S. agalactiae* showed higher susceptibility to cloxacillin (75%) than *S. dysgalactiae* (43%). A study in dairy cows conducted in Switzerland between 2011 and 2013 reported high antimicrobial susceptibility to amoxicillin-clavulanic acid (99%; 100%) and ampicillin (92%; 94%) for *S. uberis* and *S. dysgalactiae*, respectively (Ruegsegger et al. 2014). However, we found lower sensitivity percentages for amoxicillin-clavulanic acid in the region. Moreover, our findings indicated that *S. dysgalactiae* did not show susceptibility to ampicillin, and *S. uberis* showed very low susceptibility to this antibiotic. Conversely, *S. agalactiae* exhibited high susceptibility to ampicillin. According to a study, *S. agalactiae* demonstrated resistance to tetracycline (46%) and erythromycin (15%), while *S. dysgalactiae* showed resistance to tetracycline (38.5%) and erythromycin (20%) (Kabelitz et al. 2021).

While our findings are consistent with this study, we observed higher percentages of resistance in our country. The *tetL*, *tetS*, and *tetM* genes have frequently been reported in bovine mastitis to be frequently encountered in *Streptococcus* isolates (Kaczorek et al. 2017a). In several countries, including France, Brazil, Canada, Portugal, Poland, Egypt, Argentina, and China, *S. uberis*, *S. agalactiae*, or *S. dysgalactiae* have been reported to harbour the gene *tetM* in bovine mastitis (Naranjo-Lucena and Slowey 2023). Also, it has been reported that the dominance of the *tetM* gene indicates that the mechanism of resistance is mainly mediated by the protection of the ribosomes and not by the efflux pump (Rosa et al. 2021). The dominance of the *tetM* gene suggests that resistance mechanisms primarily involve ribosome protection rather than efflux pumps (Rosa et al. 2021). We detected resistance to tetracycline in our isolates, with *tetM* being the most frequently detected resistance gene in *S. uberis* (62.5%), followed by *tetS* (50%), *ermC* (43.7%), and *tetL* (40.6%). Saed and Ibrahim (2020) reported the common presence of the *ermB* gene in *Streptococcus* isolates from mastitis in dairy cows, with a prevalence of 40% among erythromycin-resistant isolates. Erythromycin-resistant *Streptococcus* isolates from sheep milk have also been reported to carry the *ermB* gene (Rosa et al. 2021). The *ermC* gene resistance was found in 11 *S. uberis* isolates and 3 *S. agalactiae* isolates. Resistance to the *ermC* gene was detected in multiple resistance groups. However, only one isolate of each strain demonstrated possessing or carrying the *ermB* gene, which is 3.1% of the samples. The *aad-6* gene was detected in *S. uberis* and *S. dysgalactiae* species in dairy cows (Kaczorek et al. 2017a). Another study reported that aminoglycoside-resistant *Streptococcus* isolates are negative for *aad-6* genes (Rosa et al. 2021). Our results indicate that only 6 *S. uberis* isolates carried the *aad-6* gene, aligning with the inherent aminoglycoside resistance of *Streptococcus* strains. In Germany, the *ermB* gene was only detected in *S. uberis*, whereas both *S. agalactiae* and *S. dysgalactiae* species harboured the *ermB* and *ermC* genes in dairy cows. However, in our study, we found the *ermB* gene only in *S. uberis* isolates. The *blaZ* gene is frequently detected in France for *S. uberis*, *lnuD* for *S. uberis*, and *S. dysgalactiae* in Poland (Naranjo-Lucena and Slowey 2023). Detection of the *blaZ* gene has also been reported in sheep milk (Rosa et al. 2021). However, we detected the *blaZ* gene in *S. uberis* isolates, and the *lnu* gene in one *S. agalactiae* isolate (3.1%), which is consistent with its reported low prevalence (Haenni et al. 2018).

In conclusion, our findings suggest that *Strep-tococcus* isolates from sheep mastitis have high antimicrobial resistance and carry various virulence genes that may be harmful to sheep. Further studies are needed to explore the epidemiology of *Streptococcus*-derived herd infections and their ability to confer resistance to antimicrobials regarding new virulence and resistance.
